# Supplementary material for: Comparative Genomics of Human- and Wastewater-Derived CPE Isolates in The Netherlands Reveals Shared and Complementary Characteristics
Source: Microorganisms. 2025 Dec 20;14(1):16. doi: 10.3390/microorganisms14010016 (PMC12844007; doi:10.3390/microorganisms14010016)
Supplement: Supplementary file 1 [file microorganisms-14-00016-s001.zip › Supplementary table S1.pdf]

Table S1. CPE isolates from human surveillance

Carbapenemase allel[1]	Reason for culture				
	Screening patient	Clinical indication	Screening worker	Not known	Total
Escherichia coli					
bla$_{IMP-4}$	1	0	0	0	1
bla$_{KPC-2}$	3	0	0	8	11
bla$_{KPC-2+}$	1	0	0	0	1
bla$_{KPC-3}$	0	0	0	1	1
bla$_{NDM-1}$	23	4	0	8	35
bla$_{NDM-1+}$	0	0	0	1	1
bla$_{NDM-19}$	0	1	0	2	3
bla$_{NDM-4}$	0	0	0	1	1
bla$_{NDM-5}$	51	16	1	68	136
bla$_{NDM-5+}$	4	0	0	3	7
bla$_{NDM-6}$	1	0	0	0	1
bla$_{NDM-7}$	5	1	0	9	15
bla$_{NDM-7+}$	0	0	0	1	1
bla$_{OXA-181}$	25	4	0	20	49
bla$_{OXA-232}$	0	0	0	1	1
bla$_{OXA-244}$	19	5	0	38	62
bla$_{OXA-427}$	0	0	0	1	1
bla$_{OXA-48}$	79	35	0	85	199
bla$_{OXA-484}$	0	0	0	1	1
bla$_{VIM-1}$	12	3	0	4	19
bla$_{VIM-2}$	1	0	0	0	1
bla$_{VIM-4}$	1	0	0	0	1
not detected	0	1	0	2	3
E. coli total	*226*	*70*	*1*	*254*	*551*
***Klebsiella* pneumoniae complex**					
bla$_{GES-5}$	0	0	0	1	1
bla$_{IMP-28}$	0	0	0	3	3
bla$_{KPC-2}$	28	10	0	44	82
bla$_{KPC-2+}$	1	0	0	1	2
bla$_{KPC-3}$	29	6	0	11	46
bla$_{KPC-3+}$	0	0	0	1	1
bla$_{NDM-1}$	77	19	0	51	147
bla$_{NDM-1+}$	17	1	0	12	30
bla$_{NDM-19}$	0	0	0	1	1
bla$_{NDM-4}$	2	0	0	0	2
bla$_{NDM-5}$	4	2	0	9	15
bla$_{NDM-5+}$	5	1	0	14	20
bla$_{NDM-7}$	5	2	0	3	10
bla$_{OXA-162}$	2	1	0	0	3

bla$_{\text{OXA-181}}$	10	4	0	16	30
bla$_{\text{OXA-232}}$	14	4	0	6	24
bla$_{\text{OXA-244}}$	1	0	0	1	2
bla$_{\text{OXA-245}}$	3	0	0	2	5
bla$_{\text{OXA-48}}$	127	49	0	117	293
bla$_{\text{VIM-1}}$	3	1	0	5	9
not detected	0	0	0	1	1
K. pneumoniae c. total	328	100	0	299	727

[1]A plus sign ('+') indicates the presence of a second carbapenemase allel, see details in Tabel S1b

Table S1b. CPE isolates with two carbapenemase alleles

	Second carbapenemase allele						
**First carbapenemase allel**	*bla*$_{\text{NDM-1}}$	*bla*$_{\text{OXA-181}}$	*bla*$_{\text{OXA-232}}$	*bla*$_{\text{OXA-244}}$	*bla*$_{\text{OXA-48}}$	*bla*$_{\text{OXA-484}}$	*bla*$_{\text{VIM-1}}$
Escherichia coli							
bla$_{\text{KPC-2}}$	1						
bla$_{\text{NDM-1}}$		1					
bla$_{\text{NDM-5}}$		5		1		1	
bla$_{\text{NDM-7}}$			1				
Klebsiella pneumoniae complex							
bla$_{\text{KPC-2}}$					1		1
bla$_{\text{KPC-3}}$	1						
bla$_{\text{NDM-1}}$			10		20		
bla$_{\text{NDM-5}}$		4	4		12		
