# Supplementary material for: Comparative Genomics of Human- and Wastewater-Derived CPE Isolates in The Netherlands Reveals Shared and Complementary Characteristics
Source: Microorganisms. 2025 Dec 20;14(1):16. doi: 10.3390/microorganisms14010016 (PMC12844007; doi:10.3390/microorganisms14010016)
Supplement: Supplementary file 1 [file microorganisms-14-00016-s001.zip › Supplementary table S2_revised.pdf]

Table S2. CPE isolates from wastewater

Species	Carbapenemase allele	Total isolates	WWTP (n=37)			Hospitals (n=6) and nursing home (n=1)		
			Nr. of isolates	Nr. of locations	Nr. of samples	Nr. of isolates	Nr. of locations	Nr. of samples
Eco	*bla*$_{KPC-2}$	30 (55)	19 (32)	3 (3)	9 (9)	11 (23)	1 (1)	4 (4)
Eco	*bla*$_{KPC-3}$	9 (26)	5 (14)	1 (1)	4 (9)	4 (12)	1 (1)	4 (7)
Eco	*bla*$_{NDM-1}$	19 (22)	11 (13)	5 (5)	8 (8)	8 (9)	3 (3)	5 (5)
Eco	*bla*$_{NDM-5}$	55 (61)	54 (60)	20 (20)	42 (43)	1 (1)	1 (1)	1 (1)
Eco	*bla*$_{NDM-7}$	1 (1)	1 (1)	1 (1)	1 (1)	0 (0)	0 (0)	0 (0)
Eco	*bla*$_{GES-5}$	11 (15)	7 (10)	1 (1)	5 (6)	4 (5)	2 (1)	2 (2)
Eco	*bla*$_{OXA-48}$	5 (6)	0 (0)	0 (0)	0 (0)	5 (6)	2 (2)	4 (5)
Eco	*bla*$_{OXA-181}$	22 (23)	22 (23)	15 (15)	20 (20)	0 (0)	0 (0)	0 (0)
Eco	All CP Eco	152 (209)	119 (153)	33 (33)	69 (70)	33 (56)	6 (6)	17 (19)
Kpn	*bla*$_{KPC-2}$	14 (23)	8 (13)	3 (3)	6 (7)	6 (10)	1 (1)	3 (4)
Kpn	*bla*$_{KPC-3}$	14 (46)	6 (32)	1 (1)	6 (22)	8[2] (14)	3[2] (3)	7[2] (9)
Kpn	*bla*$_{NDM-1}$	11 (15)	11 (15)	8 (8)	10 (12)	0 (0)	0 (0)	0 (0)
Kpn	*bla*$_{NDM-5}$	2 (2)	1 (1)	1 (1)	1 (1)	1 (1)	1 (1)	1 (1)
Kpn	*bla*$_{NDM-1}$, *bla*$_{NDM-5}$[1]	1 (2)	1 (2)	1 (1)	1 (1)	0 (0)	0 (0)	0 (0)
Kpn	*bla*$_{OXA-48}$	8 (9)	0 (0)	0 (0)	0 (0)	8 (9)	1 (1)	4 (4)
Kpn	*bla*$_{OXA-181}$	1 (1)	1 (1)	1 (1)	1 (1)	0 (0)	0 (0)	0 (0)
Kpn	All CP Kpn	51 (98)	28 (64)	12 (12)	25 (41)	23[2] (34)	4[2] (4)	13[2] (15)
Eco & Kpn	All CPE	203 (307)	147 (217)	37 (37)	77 (81)	56[2] (90)	7[2] (7)	18[2] (20)

Indicated are the total number of isolates, locations and samples included in the comparative analysis with human isolates, i.e. after removing copy isolates. Between brackets and in grey font are the total number of isolates, locations and samples that were originally available.

[1]as deduced from sanger sequences which have both an G and T at nucleotide position 262 (translating into both a Valine (GTG) and a Leucine (TTG) at amino acid position 88), and both an A and C at nucleotide position 460 (translating into both Methionine (ATG) and a Leucine (CTG) at amino acid position 154). [2]One of the isolates was from a wastewater sample from a nursing home, the remainder was obtained from hospital wastewater. Eco=*E. coli*, Kpn=*K. pneumoniae* complex.
